# Optimum threshold of the 4Kscore for biopsy in men with negative or indeterminate multiparametric magnetic resonance imaging

**DOI:** 10.1002/bco2.235

**Published:** 2023-05-01

**Authors:** Ricardo de Almeida S, Jamie Thomas, Matthew M. Mason, Maria F. Becerra, Ali Merhe, Isildinha M. Reis, Deukwoo Kwon, Nachiketh Soodana‐Prakash, Ashutosh Tewari, Vipul Patel, Vinayak Wagaskar, Badrinath Konety, Ali Kasraeian, Stefan Czarniecki, Gregory R. Thoreson, Eric H. Kim, Sanjaya Swain, Dipen J. Parekh, Sanoj Punnen

**Affiliations:** ^1^ Desai Sethi Urology Institute, Miller School of Medicine, Sylvester Cancer Center University of Miami Miami Florida USA; ^2^ School of Medicine University of Miami Miller Miami Florida USA; ^3^ Division of Biostatistics, Department of Public Health Sciences, School of Medicine University of Miami Miller Miami Florida USA; ^4^ Department of Urology Icahn School of Medicine at Mount Sinai New York New York USA; ^5^ Global Robotics Institute, Florida Hospital‐Celebration Health, College of Medicine University of Central Florida Orlando Florida USA; ^6^ Department of Urology Icahn School of Medicine at Mount Sinai Hospital New York New York USA; ^7^ Allina Health Cancer Institute Minneapolis Minnesota USA; ^8^ Kasraeian Urology Jacksonville Florida USA; ^9^ HIFU Clinic, Department of Urology St. Elizabeth Hospital Warsaw Poland; ^10^ Urology Clinics of North Texas Dallas Texas USA; ^11^ School of Medicine Washington University St. Louis Missouri USA

**Keywords:** 4Kscore, multiparametric magnetic resonance imaging, PIRADS, prostate biopsy, prostate cancer

## Abstract

**Objective:**

The study aims to identify the optimal 4Kscore thresholds to determine the need for a prostate biopsy when multiparametric magnetic resonance imaging (MRI) (mpMRI) is negative or indeterminate.

**Materials and methods:**

We analysed retrospective data from men in eight different institutions who underwent an mpMRI, 4Kscore and prostate biopsy for evaluation of prostate cancer. We selected men with a negative (PIRADS ≤2) or indeterminate (PIRADS 3) mpMRI. 4Kscore values were categorized into ranges of 1–7, 8–19, 20–32 and greater than 32. We evaluated the proportion of men with grade group 2 or higher (GG2+) cancer in groups defined by PIRADS and 4Kscore. We also evaluated the number of biopsies avoided and GG2+ cancer missed in each group reported depend on 4Kscore cutoff points.

**Results:**

Among 1111 men who had an mpMRI, 4Kscore and biopsy, 625 of them had PIRADS ≤3 on mpMRI: 374 negative (PIRADS ≤2) and 251 indeterminate (PIRADS 3). In men with a negative mpMRI, we found a 4Kscore cut‐point of 33 resulted in an increased risk of GG2+ cancer on biopsy. In patients with an equivocal lesion on mpMRI, men with a 4Kscore cutoff ≥8 had a greater risk of GG2+ cancer on biopsy. Decision curve analysis supported the proposed cut‐points in each mpMRI group.

**Conclusions:**

In men with negative and indeterminate mpMRI, we found the best 4Kscore threshold to determine the need for biopsy to be 33 and 8 respectively. Future prospective studies in independent populations are needed to confirm these findings.

## 1 INTRODUCTION

Multiparametric magnetic resonance imaging (MRI) (mpMRI) of the prostate has been shown to reduce the number of biopsies performed and indolent cancers detected, while improving the detection of clinically significant prostate cancer (csPCa).^1^, ^2^ Consequently, we have seen a dramatic rise in the use of mpMRI in the evaluation of men being referred for suspicion of prostate cancer (PCa).^3^ Nevertheless, mpMRI interpreted as negative (PIRADS ≤2) or indeterminate (PIRADS 3) presents a challenge for the clinician due the imperfect negative predictive value of mpMRI and that concern for missing a clinically significant cancer.^4^ To address this scenario, novel screening methods using a combination of mpMRI and biomarkers have shown increasing sensitivity and accuracy compared with mpMRI alone.^5^, ^6^

Use of the 4Kscore, a risk prediction tool combining a 4‐kallikrein (4K) panel with clinical features, has been demonstrated to improve the diagnosis of PCa.^7^ Previous studies have shown that a combination of mpMRI and 4Kscore outperformed either test alone for deciding on the need for a prostate biopsy.^5^ A 4Kscore cutoff of 7.5 has been previously proposed to select men at low risk for csPCa, but these results were based on men without mpMRI.^8^ The best 4Kscore threshold for deciding on a biopsy when combined with mpMRI remains unclear.

In this study, we evaluated a large multi‐institutional dataset to identify the ideal 4Kscore threshold to determine the need for a prostate biopsy in men with a negative or indeterminate mpMRI.

## 2 MATERIALS AND METHODS

### 2.1 Patient selection

We retrospectively evaluated men from eight different institutions (University of Miami, Mount Sinai NY, Washington University, University of Minnesota, Kasraeian Urology, Advent Health Global Robotics Institute, HIFU Clinic Poland, and Urology Clinics of North Texas) seen in a clinic between 2013 to 2018. Only patients with mpMRI, 4Kscore and prostate biopsy were included in our analysis. In most cases, the mpMRI and 4Kscore test were done concurrently and both were always completed before a prostate biopsy. All mpMRIs were performed on a 3T magnet using a surface array coil with diffusion weighted and dynamic contrast enhanced images, which were interpreted by fellowship trained radiologists using the version of Prostate Imaging Reporting and Data System (PIRADS) that was current at that time. We selected men who were found to have negative or indeterminate mpMRIs. ‘Negative mpMRI’ was defined as a PIRADS ≤2 and ‘indeterminate mpMRI’ was defined as a PIRADS 3.

4KScore values were categorized into ranges of 1–7, 8–19, 20–32, and greater than 32. The use of 4Kscore categories, rather than continuous values, was required by the study's central IRB to maintain patient confidentiality. Histopathology of biopsy cores were interpreted according to the standard of care at their respective institution, which included fellowship trained pathologists at most sites.

### 2.2 Statistical analysis

The primary endpoint for this analysis was the detection of csPCa, defined as grade group 2 or higher (GG2+) cancer on prostate biopsy. Logistic regression was used to evaluate the association between PIRADS and 4Kscore results and the likelihood of having a csPCa on biopsy of the prostate. The proportion of men with grade group 2 or higher (GG2+) cancer in groups defined by mpMRI (negative and indeterminate) and categories of 4Kscore was evaluated and the number of biopsies avoided and csPCa missed in each group reported. Decision curve analysis (DCA) was performed to evaluate the benefits in clinical utility of different 4K threshold values for deciding on the need for a biopsy in each mpMRI group.^9^ *χ* ^2^ test was used to compare categorical study variables. Statistical analyses were performed with SAS version 9.4 and R software dca. R package. All tests were two‐sided and statistical significance was considered when *p* < 0.05.

## 3 RESULTS

This study evaluated 1111 men who were referred for PCa evaluation and underwent mpMRI and 4Kscore prior to biopsy of the prostate. Among these men, 625 had a negative or indeterminate mpMRI: 374 (60%) PIRADS ≤2, and 251 (40%) with PIRADS 3 region of suspicion on mpMRI, respectively. Nearly one third of the cohort (201 men) had PCa of which 87 (43%) were csPCa. In this cohort, 33% were found to have a 4Kscore ≥20. The demographics and clinical characteristics of the patients by mpMRI findings are shown in Table 1.

**TABLE 1:** Patient characteristics overall and by result of mpMRI.

			mpMRI result				
Variable	All patients		PIRADS ≤2		PIRADS 3		*p*
	*N*	%	*N*	%	*N*	%	
All	625	100.0	374	100.0	251	100.0	—
Age, in years							
<45	3	0.5	2	0.5	1	0.4	0.904
45–75	587	93.9	350	93.6	237	94.4	
>75	35	5.6	22	5.9	13	5.2	
DRE							
Abnormal	121	19.4	86	23.0	35	13.9	0.003
Normal	439	70.2	258	69.0	181	72.1	
Unknown	65	10.4	30	8.0	35	13.9	
PSA							
Mean (*SD*)	6.8	(5.6)	6.8	(6.1)	6.9	(4.6)	0.827
Prior biopsy							
No	380	60.8	237	63.4	143	57.0	0.108
Yes	245	39.2	137	36.6	108	43.0	
4Kscore							
1–7	179	28.6	104	27.8	75	29.9	0.902
8–19	238	38.1	146	39.0	92	36.7	
20–32	96	15.4	56	15.0	40	15.9	
33–95	112	17.9	68	18.2	44	17.5	
Results of the biopsy (GG)							
Negative	424	67.8	272	72.7	152	60.6	<0.001
GG1 (GS ≤6)	114	18.2	66	17.6	48	19.1	
GG2 (GS 3 + 4)	54	8.6	24	6.4	30	12.0	
GG3 (GS 4 + 3)	16	2.6	3	0.8	13	5.2	
GG4 (GS 8)	10	1.6	5	1.3	5	2.0	
GG5 (GS 9 & 10)	7	1.1	4	1.1	3	1.2	

Abbreviations: mpMRI, multiparametric magnetic resonance imaging; PSA, prostate‐specific antigen.

Table 2 depicts the rates and the 95% confidence intervals for PCa classified as GG2 or higher cancer in mpMRI negative or indeterminate patients by 4Kscore ranges. Among men with a negative mpMRI, those with a 4Kscore of 33 or greater had a higher risk of csPCa compared with those in the lower 4Kscore ranges. Among those with indeterminate mpMRI, those with a 4Kscore below 8 had lower risk of csPCa compared with those in the higher 4Kscore ranges. Thus, among those with indeterminate mpMRI, those with a 4Kscore 8 or greater had higher risk of csPCa compared with those with 4Kscore 1–7.

**TABLE 2:** Observed rates and 95% confidence intervals of GG2 or higher cancer by 4Kscore in patients with negative (PIRADS ≤2) or indeterminate (PIRADS 3) multiparametric magnetic resonance imaging.

PIRADS	4K 1–7	4K 8–19	4K 20–32	4K 33–95
≤2	1/104 (1.0%)	9/146 (6.2%)	5/56 (8.9%)	21/68 (30.9%)
	(0%, 5.2%)	(2.9%, 11.4%)	(3%, 19.6%)	(20.2%, 43.3%)
3	3/75 (4.0%)	20/92 (21.7%)	9/40 (22.5%)	19/44 (43.2%)
	(0.8%, 11.2%)	(13.8%, 31.6%)	(10.8%, 38.5%)	(28.3%, 59.0%)

*Note*: Percentage or a 95% confidence interval by the Clopper–Pearson method are shown inside parentheses.

We explored these thresholds for performing a biopsy and evaluated the number of biopsies avoided and cancers missed. In patients with a negative mpMRI, initiating a biopsy at a 4Kscore cutoff of 33 resulted in 82% (306/374) of biopsies to be avoided. Among these men who would have avoided a biopsy, 15 (5%) had GG2+ cancer. These 15 men represented 42% (15/36) of men with GG2+ in this cohort; however, only 2 (13%) of them had GG3 or higher cancer. Alternatively, using a 4Kscore cutoff of 8 allowed only 3% (1/36) of GG2+ cancers to be missed; however, 54% (202/374) more men would undergo unnecessary biopsy (Table 3).

**TABLE 3:** Biopsies avoided and cancers missed based on 4Kscore threshold for biopsy in patients with a negative (PIRADS ≤2) and indeterminate (PIRADS 3) multiparametric magnetic resonance imaging.

4Kscore cutoff (≥ *x*)	Biopsies performed	Biopsies avoided	GG1 (GS6) missed	GG2+ missed	GG3+ missed
PIRADS ≤2 (*N* = 374)	*N* = 374	*N* = 374	*N* = 66	*N* = 36	*N* = 12
8	72.2% (67.3%, 76.7%)	27.8% (23.3, 32.7%)	14	2.8% (0.1%, 14.5%)	1
20	33.2% (28.4%, 38.2%)	66.8% (61.8%, 71.6%)	40	27.8% (14.2%, 45.2%)	2
33	18.2% (14.4%, 22.5%)	81.8% (77.5%, 85.6%)	51	41.7% (25.5%, 59.2%)	2
PIRADS 3 (*N* = 251)	*N* = 251	*N* = 251	*N* = 48	*N* = 51	*N* = 21
8	70.1% (64.0%, 75.7%)	29.9% (24.3%, 36.0%)	12	5.9% (1.2%, 16.2%)	1
20	33.5% (27.7%, 39.7%)	66.5% (60.3%, 72.3%)	32	45.1% (31.1%, 59.7%)	8
33	17.5% (13.0%, 22.8%)	82.5% (77.2%, 87.0%)	37	62.8% (48.1%, 75.9%)	11

*Note*: 95% confidence intervals by the Clopper–Pearson method are shown in parentheses.

In patients with a PIRADS 3 lesion, initiating a biopsy using a 4Kscore cutoff of 8 resulted in 75 (30%) men avoiding a biopsy of which 3 men would have had a missed GG2+ cancer. This corresponds to missing only 6% (3/51) of all GG2+ cases, of which only 1 had GG3+ cancer. Alternatively, using a 4Kscore cutoff of 20 resulted in 167 (67%) men avoiding a biopsy of which 23 would have had GG2+ cancers missed. This corresponds to a missing 45% (23/51) of all GG2+ cases, of which 35% (8/23) were GG3+ (Table 3).

**FIGURE 1:**
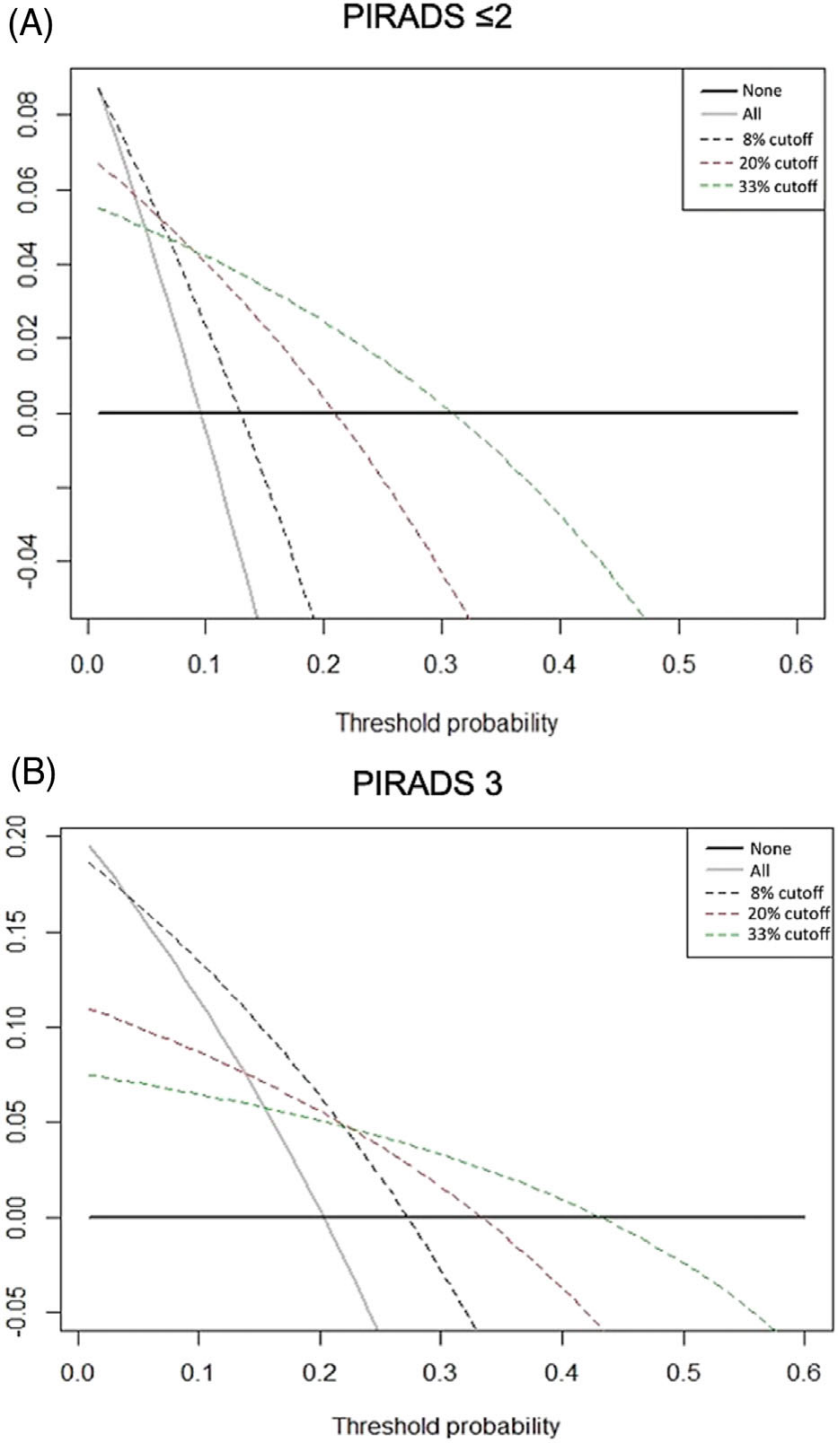
Decision curve analyses (DCA) comparing net benefit of various 4Kscore thresholds in men with negative (PIRADS ≤2) and indeterminate (PIRADS 3) multiparametric magnetic resonance imaging mpMRI. DCA shows that in men with a negative mpMRI, a 4Kscore cutoff of 33 for deciding on a biopsy had the highest net benefit across all reasonable threshold probabilities for performing a biopsy. For men with an indeterminate mpMRI, a 4Kscore cutoff of 8 for deciding on a biopsy had the highest net benefit, until a threshold probability of 25% or higher for performing a biopsy, where the 33 cutoff had the greatest net benefit.

DCA showed the highest net benefit for using a 4Kscore cutoff of 33 across a threshold probability above 15% in men with a negative mpMRI (Figure 1A). For men with an indeterminate mpMRI using a 4Kscore threshold of 8 had the highest net benefit below a threshold probability of 25%; however, above that, using a 4Kscore threshold of 33 to decide on biopsy had the highest net benefit (Figure 1B).

Table S1 provides the probability of GG2+ cancer with 95% confidence intervals for each mpMRI group and 4Kscore category stratified by prior biopsy status. Due to the low numbers in each group the confidence intervals between various 4Kscore categories overlapped. However, among men who were biopsy naïve, those with a 4Kscore below 8 had a lower risk of GG2+ cancer compared with those who had a 4Kscore above 33 for both the mpMRI negative and indeterminate groups.

## 4 DISCUSSION

Although mpMRI is increasingly being used in the evaluation of men with suspicion of prostate cancer, there is variability in the management of men with indeterminate findings and concern about missed cancer in those with a negative mpMRI. Several molecular biomarkers are currently available to evaluate the risk of csPCa, and many of them have validated cutoffs to support recommending a biopsy of the prostate.^10^ Recent studies have shown that combining mpMRI and biomarkers can enhance the selection of patients for biopsy compared with either test alone.^5^ The current literature, however, is unclear on appropriate cutoff values for biomarkers to decide on the need for biopsy in men with negative or equivocal (PIRADS 3) results. Determining these values will optimize the use of mpMRI and biomarkers in biopsy decision making, helping to achieve a more delicate balance between detecting clinically significant disease while avoiding an unnecessary and risk‐prone procedure.

This multi‐institutional study of 625 men with negative or equivocal mpMRI retrospectively assessed the use of three different 4Kscore cutoff values. In men with a negative mpMRI, we found a 4Kscore cutoff of 33 resulted in an increased risk of GG2+ cancer on biopsy with confidence intervals that failed to overlap with those using lower 4Kscore cutoffs. This suggests that in patients with a negative mpMRI, a 4Kscore ≥33 should suggest the need for prostate biopsy due to increased risk of csPCa. Alternatively, men with a score below 33 had a less than 10% risk of csPCa with overlapping confidence intervals between the groups suggesting no appreciable difference in csPCa risk. In patients with an equivocal lesion on mpMRI, we found that men with a 4Kscore cutoff greater than 8 had a greater than 20% risk of csPCa with confidence intervals that failed to overlap with those who had a 4Kscore below 8, who had a less than 5% of csPCa. This suggests that patients with an equivocal mpMRI and a 4Kscore less than 8 may be able to safely avoid biopsy due to a low risk of csPCa, whereas those with a 4Kscore above 8 have a significantly increased risk of csPCa and may want to consider biopsy. These thresholds were supported by DCA but require further validation in an independent cohort.

In our cohort of men with a negative mpMRI, using a 4Kscore of 8 as a cutoff for initiating prostate biopsy would have allowed for 82% (306/374) of biopsies to be avoided. This includes deferring prostate biopsy in 77% (51/66) of those with an indolent cancer and 42% (15/36) with a clinically significant cancer, of which only 13% (2/15) were GG3+ cancers. If a cutoff value of 20 was instead used, less GG2 cancers would be missed (8% vs. 13%), but no additional GG3+ cancers would be detected and a significantly lower number of biopsies would be avoided (67% vs. 82%), including the detection of 11 (17%) additional indolent cancers. In men with an equivocal mpMRI, the use of 8 as a threshold value would have allowed for 30% (75/251) of biopsies to be avoided but would miss only 6% (3/51) of clinically significant cancers. While the use of 20 as a cutoff would allow for more biopsies to be deferred (67% vs. 30%), this would result in 45% (23/51) of GG2+ cancers to be missed, of which 35% (8/23) were GG3+ cancers.

Although the current study evaluated 4Kscore cut‐points for conducting a biopsy after the mpMRI was negative or equivocal, we appreciate that many providers may begin evaluation of elevated prostate‐specific antigen (PSA) with a biomarker. These results suggest that if a 4Kscore of >33 is found, the patient would require a prostate biopsy regardless of the mpMRI results. In such cases an mpMRI would only be necessary for biopsy targeting. If a 4Kscore value is <33 is found, then an mpMRI would allow for further triaging of patients who would benefit from a prostate biopsy versus those in whom a biopsy could be avoided.

Current practice models typically suggest a prostate biopsy be performed in those patients with a PIRADS 3 lesion, making up 22%–32% of those tested with mpMRI.^11^ However, only 16%–21% of these patients will be found to have csPCa, and thus, there is a significant potential to improve risk stratification in this cohort.^11^ A combination screening method using mpMRI and PC biomarkers offers a promising solution to this problem. Tosoian et al. conducted a similar study to ours using the urinary MyProstateScore (MPS), concluding that MPS significantly outperforms PSA and PSA‐density testing in ruling out GG2+ cancer in men with equivocal mpMRI results.^12^ Other studies outside our own have examined the benefits of combining mpMRI and 4Kscore in various combinations.^13^, ^14^ However, to our knowledge, this is the first study to evaluate the best 4Kscore threshold for considering a biopsy of the prostate in men with a negative or equivocal mpMRI. Given the increasing use of mpMRI and biomarkers in prostate cancer evaluation, this study provides valuable information to guide providers on how to best utilize these tools in combination.

There are some limitations to our study that need to be acknowledged. This was a multi‐institutional retrospective cohort analysis evaluating patients who underwent evaluation for PCa in high‐volume referral centres, and thus, variations in clinical and pathology review protocols across institutions may exist. However, these conditions represent real‐world variations and yield findings that are generalizable to most urologic practices. Second, to de‐identify patient information, we were limited to assessing a patient's 4Kscore using four ranges of incremented risk rather than a continuous numerical score. This was required by the study's IRB to maintain patient confidentiality but hinders our ability to assess specific cutoff values. Another limitation of this study was that the decision to perform a biopsy was not made using a standard protocol but instead on the clinical discretion of the treating physicians. Finally, we analysed the performance of mpMRI and the 4Kscore using biopsy as the gold standard, a procedure that has known sampling errors when compared with radical prostatectomy, and this may have resulted in missed cancer. This study also has several strengths. To our knowledge, this is the first investigation to suggest 4Kscore cutoff values to optimize a patient's selection for prostate biopsy in men with negative and equivocal mpMRI's. Finally, this study's multi‐institutional nature combined with its large sample size of over 600 patients allows these data to be generalizable to all men.

Data from a multi‐institutional dataset of patients who underwent mpMRI, 4Kscore and prostate biopsy suggest that when the mpMRI results are negative, the best 4Kscore threshold to decide on the need for a biopsy is 33. However, when the mpMRI results are indeterminate (PIRADS 3), a 4Kscore of 8 is the best cutoff for deciding on the need for a biopsy. Future prospective studies in independent populations are needed to confirm these findings.

## AUTHOR CONTRIBUTIONS

Ricardo de Almeida performed conceptualization, methodology and writing of the original draft, review and editing. Jamie Thomas, Matthew Mason and Maria Becerra helped in writing the original draft, review and editing. Ali Merhe, Ashutosh Tewari, Vipul Patel, Vinayak Wagaskar, Badrinath Konety, Ali Kasraeian, Stefan Czarniecki, Gregory Thoreson, Eric Kim, Sanjaya Swain and Dipen Parekh contributed in writing review and editing. Isildinha Reis and Deukwoo Kwon performed data curation and formal analysis. Sanoj Punnen performed conceptualization, investigation, methodology, project administration and writing review and editing.

## CONFLICT OF INTEREST STATEMENT

The authors have no conflict of interest to disclose.

## Supporting information


**Table S1.** Observed rates of GG2 + or higher cancer for subgroups defined by mpMRI, 4Kscore, and prior biopsy status.
