# Supplementary material for: Optimum threshold of the 4Kscore for biopsy in men with negative or indeterminate multiparametric magnetic resonance imaging
Source: BJUI Compass. 2023 May 1;4(5):591–6. doi: 10.1002/bco2.235 (PMC10447206; doi:10.1002/bco2.235)
Supplement: Supplementary file 1 — Table S1. Observed rates of GG2 + or higher cancer for subgroups defined by mpMRI, 4Kscore, and prior biopsy status. [file BCO2-4-591-s001.docx]

**Supplemental Table 1.** Observed rates of GG2+ or higher cancer for subgroups defined by mpMRI, 4Kscore, and prior biopsy status

| **Biopsy-naïve (no prior biopsy) (N=380)** | | | | |
| --- | --- | --- | --- | --- |
| **PIRADS \ 4K** | **1-7** | **8-19** | **20-32** | **33-95** |
| **≤2** | 1/36 (2.8%)  (0.1%, 14.5%) | 8/104 (7.7%)  (3.4%, 14.6%) | 3/42 (7.1%)  (1.5%, 19.5%) | 19/55 (34.5%)  (22.2%, 48.6%) |
| **3** | 0/19 (0.0%)  (0%, 17.6%*) | 15/61 (24.6%)  (14.5%, 37.3%) | 8/30 (26.7%)  (12.3%, 45.9%) | 16/33 (48.5%)  (30.8%, 66.5%) |
| **Negative prior biopsy (N=245)** | | | | |
| **PIRADS \ 4K** | **1-7** | **8-19** | **20-32** | **33-95** |
| **≤2** | 0/68 (0%)  (0%, 5.3%*) | 1/42 (2.4%)  (0.1%, 12.6%) | 2/14 (14.3%)  (1.8%, 42.8%) | 2/13 (15.4%)  (1.9%, 45.4%) |
| **3** | 3/56 (5.4%)  (1.1%, 14.9%) | 5/31 (16.1%)  (5.5%, 33.7%) | 1/10 (10.0%)  (0.3%, 44.5%) | 3/11 (27.3%)  (6.0%, 61.0%) |
| ( ): Percentage and corresponding 95% confidence interval by the Clopper-Pearson method.  * One-sided 97.5% confidence interval. | | | | |
